# Identification and validation of a prognostic 9-genes expression signature for gastric cancer

**DOI:** 10.18632/oncotarget.17764

**Published:** 2017-05-10

**Authors:** Zhiqiang Wang, Gongxing Chen, Qilong Wang, Wei Lu, Meidong Xu

**Affiliations:** ^1^ Endoscopy Center and Endoscopy Research Institute, Zhongshan Hospital, Fudan University, Shanghai, China; ^2^ Department of General Surgery, Tongren Hospital, Shanghai Jiao Tong University School of Medicine, Shanghai, China; ^3^ The Laboratory Center of Medical School of Hangzhou Normal University, Hangzhou, China; ^4^ Department of General Surgery, Xinhua Hospital, Shanghai JiaoTong University, School of Medicine, Shanghai, China; ^5^ Institute of Biliary Tract Diseases, Shanghai JiaoTong University School of Medicine, Shanghai, China

**Keywords:** gastric cancer, prognostic model, survival analysis, clustering analysis

## Abstract

Gastric cancer (GC) is a common malignant tumor with high incidence and mortality. Reasonable assessment of prognosis is essential to improve the outcomes of patients. In this study, we constructed and validated a prognostic gene model to evaluate the risks of GC patients. To identify the differentially expressed genes between GC patients and controls, we extracted Gene expression profiles of GC patients (N=432) from Gene Expression Omnibus database and then stable signature genes by using Robust likelihood-based modeling with 1000 iterations. Unsupervised hierarchical clustering of all samples was performed basing on the characteristics of gene expressions. Meanwhile, the differences between the clusters were analyzed by Kaplan Meier survival analysis. A 9-genes model was obtained (frequency = 999; p=1.333628e^-18^), including two negative impact factors (*NR1I2* and *LGALSL*) and 7 positive ones (*C1ORF198, CST2, LAMP5, FOXS1, CES1P1, MMP7 and COL8A1*). This model was verified in single factor survival analysis (p=0.004447558) and significant analysis with recurrence time (p=0.001474831) by using independent datasets from TCGA. The constructed 9-genes model was stable and effective, which might serve as prognostic signature to predict the survival of GC patients and monitor the long-term treatment of GC.

## INTRODUCTION

Gastric cancer (GC) is the third leading cause of cancer-related death worldwide [1]. According to the report of World Health Organization (WHO), there have been more than 930,000 new cases of gastric cancer yearly in the world early in 2008 [2]. It is common that most patients were diagnosed in advanced stage of GC only. And many patients are suffering from the metastatic recurrences even after curative resection [3]. Although several improvements have been achieved in the surgical treatment of gastric cancer, the overall 5-year survival rate for all diagnosed patients is only 24.5 % in Europe [4] and 40 %-60 % in Asia [5, 6], resulting from high recurrence rate. It is reported that without receiving chemotherapy, the survival median of patients at late stage GC is less than 6 months [7]. Moreover, even the prognoses of patients with timely treatments are poor and worrying [8].

Epigenetic alterations, especially aberrant DNA methylation, and microRNA (miRNA) expression play a central role in many cancers [9–11]. In many studies on the molecular mechanism of GC, many oncogenes and tumor suppressors have been identified playing important roles in tumorigenesis of GC. The recent identifications of the new biomarkers and therapeutic targets in GC have supplied well to improve diagnosis and treatment of GC. However, in terms of the high reoccurrence rate and poor survival rate of GC, there are no commonly accepted biomarkers being established to facilitate the comprehensive management of patients, especially the prognostic prediction.

As to GC, apart from the disease staging, Lauren’s classification is another accurate well-recognized class-ification system, subdividing GC as two histomorphologic subtypes – “intestinal-differentiated” and “diffuse-undifferentiated” [12]. However, even Lauren’s classification fails to accurately guide patient therapy and functions well when predicting the outcomes of GC patients. Thus, identifying tumor markers or constructing feature gene models are still the focus of many researches and studies.

In this study, gene expression profiles of gastric cancer were analyzed to figure out the key genes affecting the prognosis of patients. With repeated Kaplan Meier survival analysis and Robust likelihood-based modeling, a 9-genes expression signature was finally identified serving as prognostic model for gastric cancer. The stability and effectiveness were verified by an extra dataset in the Cancer Genome Atlas (TCGA). The 9-gene prognostic model can function as effective prognostic tool to identify groups of patients at risk of relapse or metastasis. Besides, this might also be useful to monitor cancer survivors following treatment.

## RESULTS

### Data source

In total, 17418 expression profiles of 432 formalin fixed paraffin embedded tumor tissues of GC patients were obtained in dataset GSE26253.

**Figure 1:**
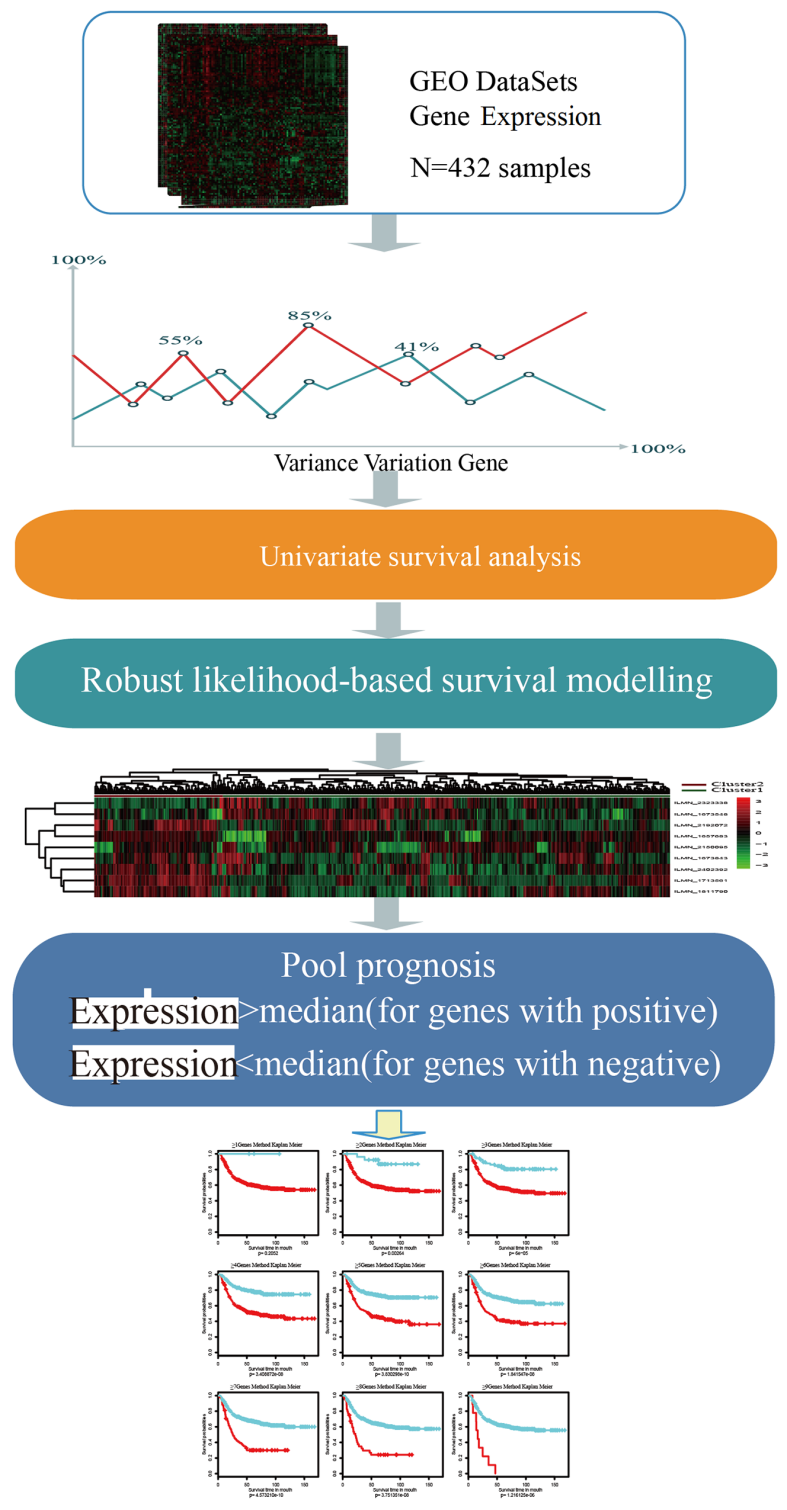
Schematic diagram for a multi-step strategy to identify gene signature for prognosis in gastric cancer Robust likelihood-based survival model with 1000 iterations were constructed for selection of stable feature. After that, genes were defined as positive-impacting factors with expression higher than median (genes with positive) and negative-impacting factors with expression lower than median (genes with negative). This process is basing on the expressing patterns in the clustering analysis to go on with survival analysis.

Schematic diagram for a multi-step strategy to identify gene signature for prognosis in GCwas listed in Figure 1. The results for each step were summarized.

### Screening of prognostic genes

A total of 11420 differentially expressed genes were identified, including 17418 probes expressing in 432 patient samples. Single-factor survival analysis identified 798 probes with significantly differential expressions (p < 0.05). Hereinto, the top 20 significantly differentially expressed genes were listed in Table 1.

**Table 1:** Top 20 significantly differential expression genes

Probe	Gene symbol	Cox p value
ILMN_1713561	C20ORF103	1.48E-09
ILMN_1811790	FOXS1	1.65E-07
ILMN_1736078	THBS4	6.59E-07
ILMN_1732158	FMO2	9.81E-07
ILMN_2402392	COL8A1	1.36E-06
ILMN_1673843	CST2	2.59E-05
ILMN_1749846	OMD	3.31E-05
ILMN_1677636	COMP	4.31E-05
ILMN_1774350	MYOZ3	7.75E-05
ILMN_2093500	ZBED5	8.06E-05
ILMN_1759792	CLIP4	0.000103058
ILMN_2138589	MERTK	0.00011729
ILMN_1735996	NOX4	0.000129726
ILMN_1782329	HIST1H4L	0.000130899
ILMN_1695079	ZNF101	0.000145513
ILMN_1693597	ZNF287	0.000208302
ILMN_1673548	HSPC159	0.00020906
ILMN_1753524	HIST1H2AB	0.000210925
ILMN_2382679	REG3A	0.000231767
ILMN_1769168	ARL10	0.000235353

One thousand Robust likelihood-based results showed that the frequency of a 9-gene feature was 999, suggesting the 9-gene as a prognostic feature. One random result of the Robust likelihood-based survival analysis was in Table 2.

**Table 2:** Survival-associated gene signature screening using forward selection

Gene ID	nloglik	AIC	Gene symbol
ILMN_2323338	644.17	1290.35*	NR1I2
ILMN_1673843	632.73	1269.46*	CST2
ILMN_1713561	627.89	1261.77*	LAMP5
ILMN_2192072	626.6	1261.2*	MMP7
ILMN_1673548	621.82	1253.65*	LGALSL
ILMN_2150095	617.24	1246.49*	CES1P1
ILMN_2402392	615.38	1244.76*	COL8A1
ILMN_1811790	614.94	1245.88*	FOXS1
ILMN_1657683	613	1244.01*	C1ORF198
ILMN_1735996	612.83	1245.65	
ILMN_1767665	611.84	1245.69	
ILMN_1732158	611.01	1246.02	
ILMN_1736078	610.86	1247.71	
ILMN_1685433	610.82	1249.63	
ILMN_2387995	610.75	1251.49	
ILMN_1748283	608.79	1249.59	
ILMN_2382705	608.48	1250.97	
ILMN_2118129	607.4	1250.8	
ILMN_1749846	606.15	1250.3	

### Multivariate survival analysis of prognostic genes

**Figure 2:**
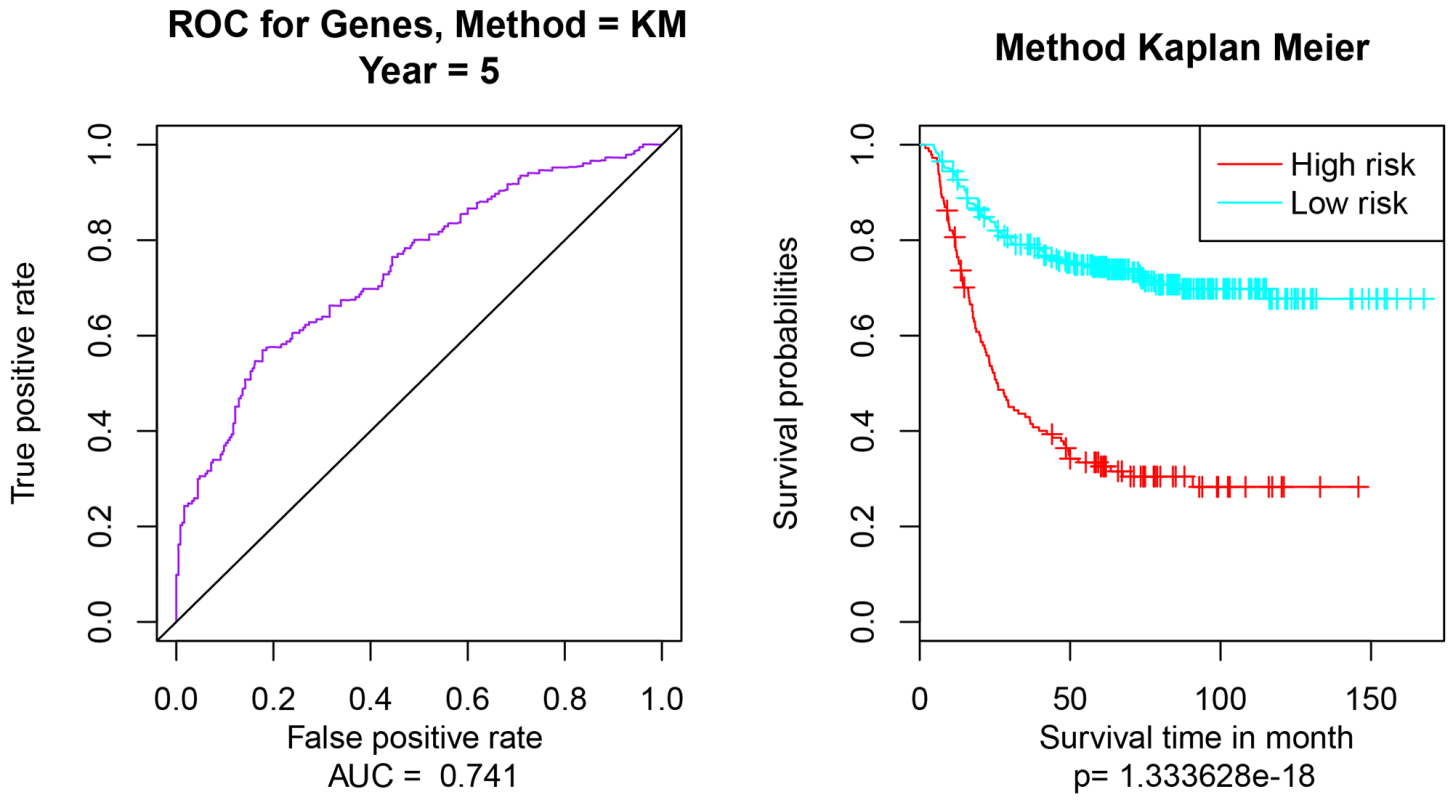
Multivariate survival analysis of 9-gene feature **(A)** the AUC curve for 9 genes, AUC = 0.741; **(B)** Kaplan Meier survival analysis of the high risk and low risk samples. The applied method is Kaplan Meier (Method = KM).

Multivariate survival analysis of 9 prognostic genes was performed to check the effect of the overall genes on the prognosis of GC. Then according to the area under the ROC curve (AUC) values and Pearson’s correlations, Kaplan Meier survival analysis was performed to estimate the differences of survival outcomes between groups (Figure 2). Multivariate survival analysis indicated that the classification of the 9-gene feature was feasible, the AUC value was 0.741 (95% confidence interval, Figure 2A) and the significant p value was 1.333628e-18 (Figure 2B).

### Clustering analysis of prognostic genes

**Figure 3:**
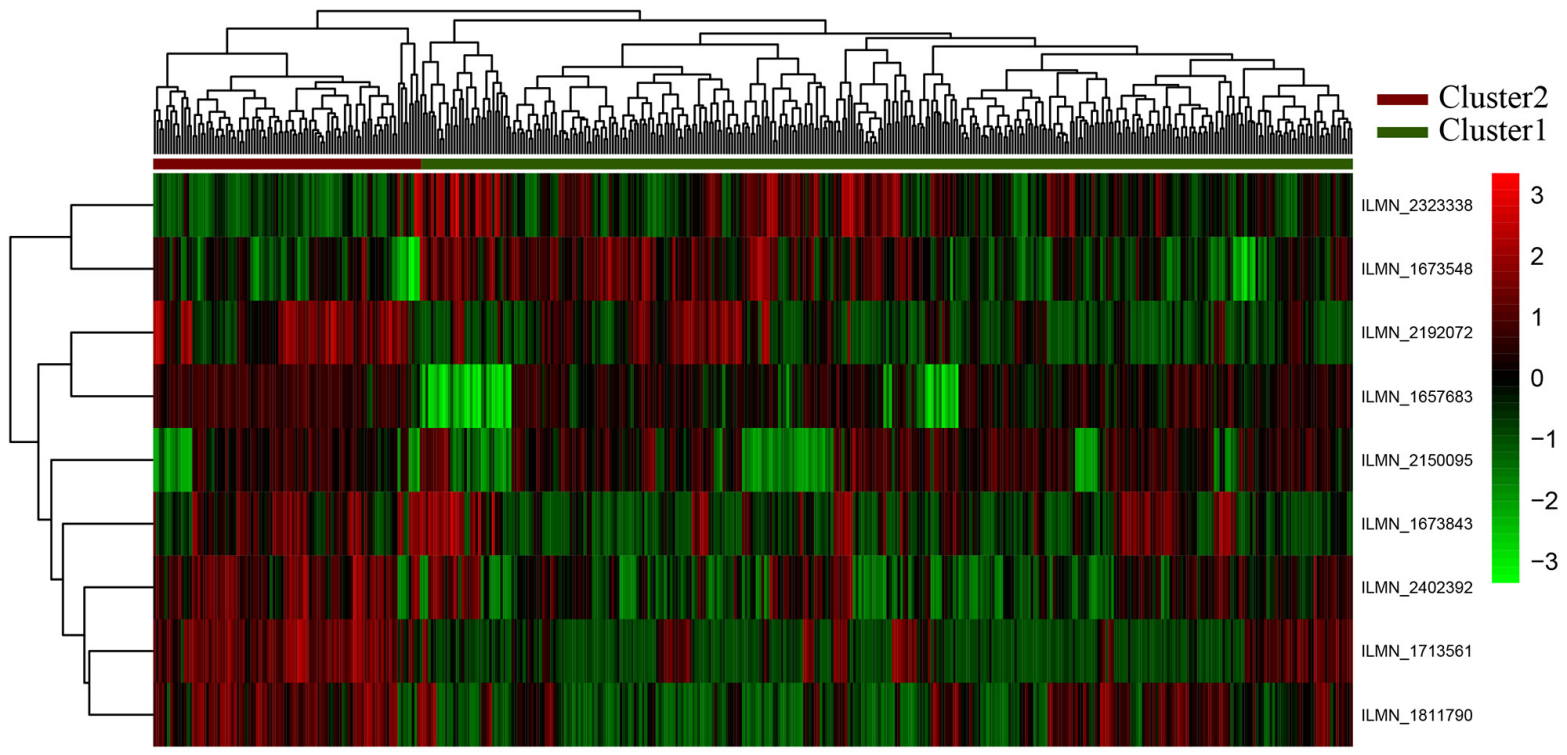
Clustering analyses for nine genes The horizontal axis above represents the samples, using Euclidean distance; The vertical right axis is the feature gene with Pearson correlation coefficient. According to the first sample categorical attribute in the spreadsheet, the samples could be grouped into two clusters. The “high risk of GC” are shown as red (Cluster 1) and the “Normal” samples are shown as green (Cluster 2).

The expression profiles of 9-gene feature were analyzed in unsupervised hierarchical clustering (Figure 3). This feature can cluster the samples into Cluster1 and Cluster2, including 336 and 96 samples, respectively.

**Figure 4:**
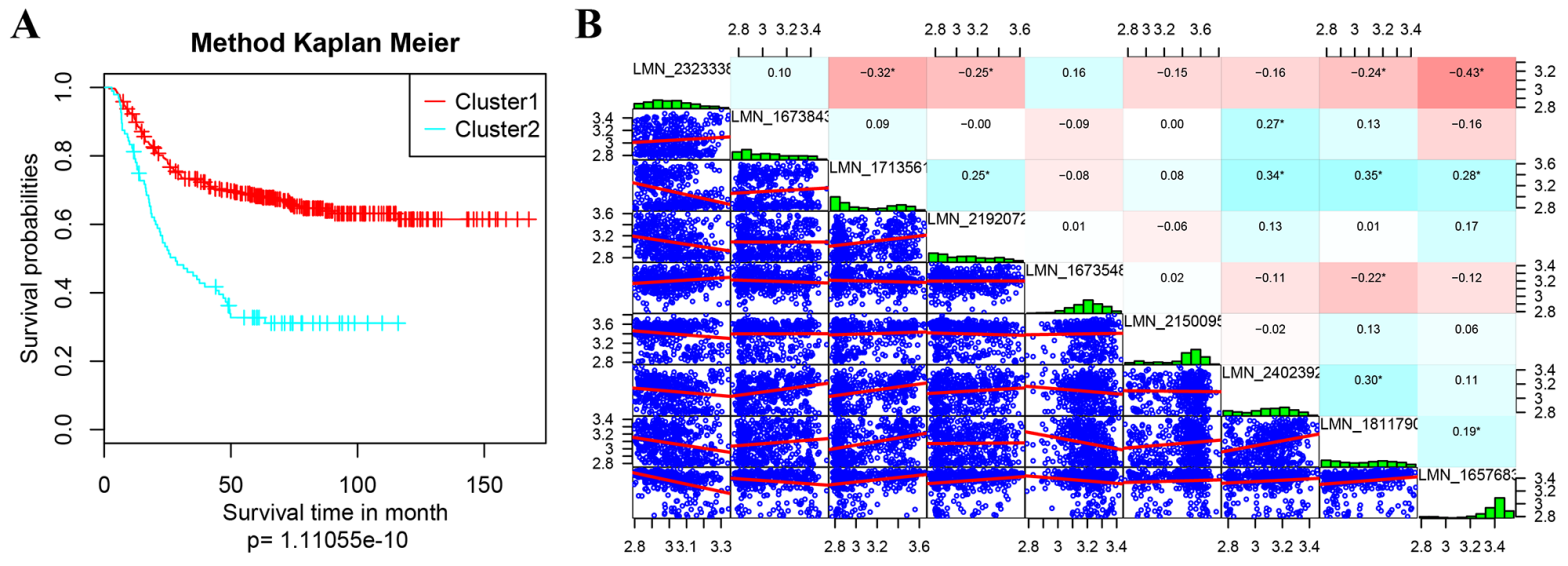
Kaplan Meier survival analyses on the prognostic differences **(A)** Kaplan Meier survival analyses of Cluster1 and Cluster2; **(B)** expression correlation analyses of 9 feature genes. In B, the diagonal were expression distribution histogram of each genes with the name marked in its rectangular box; the lower left corner is the gene expression level of the scatter diagram between two corresponding genes; the upper right corner part is the correlation coefficient of every two genes, the red represents the correlation coefficient -1, the blue represents the correlation coefficient +1;.

Kaplan Meier survival analysis on the prognostic differences of Cluster1 and Cluster2 were performed and shown in Figure 4. The prognosis of patients in Cluster1 and Cluster 2 were significantly different, suggesting the expressions of these nine genes can effectively distinguish the high- and low- risk of clinical patients (Figure 4A). Calculating the expression correlation of 9 genes, most of the genes were low (Figure 4B). It indicated that there were less overlapping of the information carried by these genes, showing low redundancy.

### Construction of prognostic model

According to the classification, the impact factors of each sample was calculated, the activated was marked as 1, or, was 0.

**Figure 5:**
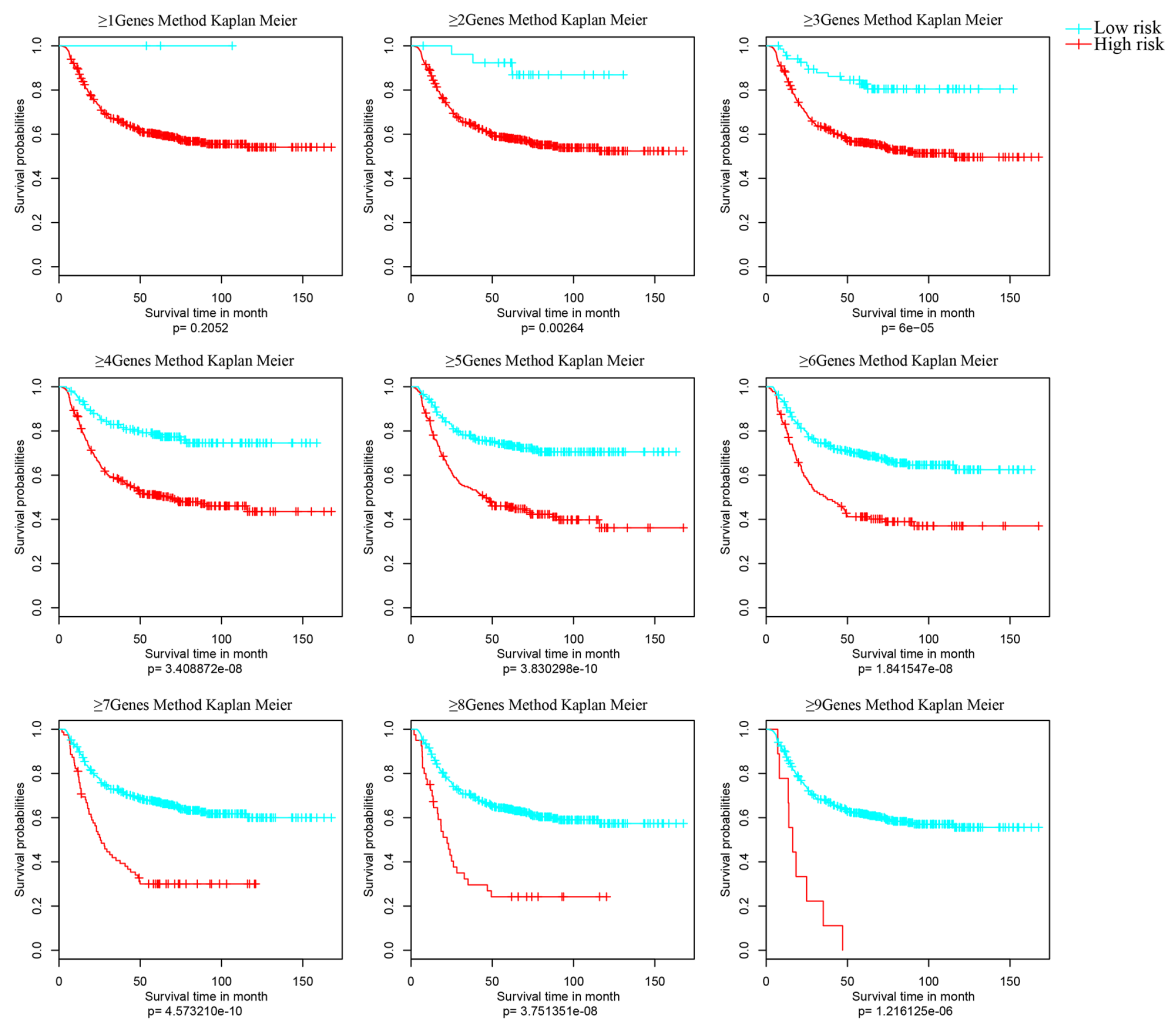
Kaplan Meier survival analyses of different clusters Samples were grouped basing on the activated impact factors of every sample (≥1, ≥2, ≥3, ≥4,…>9) into high risk (curve in red) and low risk (curve in blue). And significant p value of the corresponding cluster was obtained in Kaplan Meier univariate survival analysis. The survival time is calculated by month. The threshold is p value < 0.05.

Samples were grouped again basing on the activated impact factors of every sample (≥1, ≥2, ≥3, ≥4…). And Kaplan Meier univariate survival analysis was performed to analyze the differences of each group with significant p value (Figure 5). In Figure 4, the prognostic differences of nine classifications were all significant, especially the classification model with no less than 5 genes (≥5Genes).

Finally, the ≥5Genes cluster model was identified as the final model of the 9-gene prognostic feature. That is, patient with five genes of the 9-genes prognostic feature identified activated was suggested as high risk.

### Validation of prognostic model

**Figure 6:**
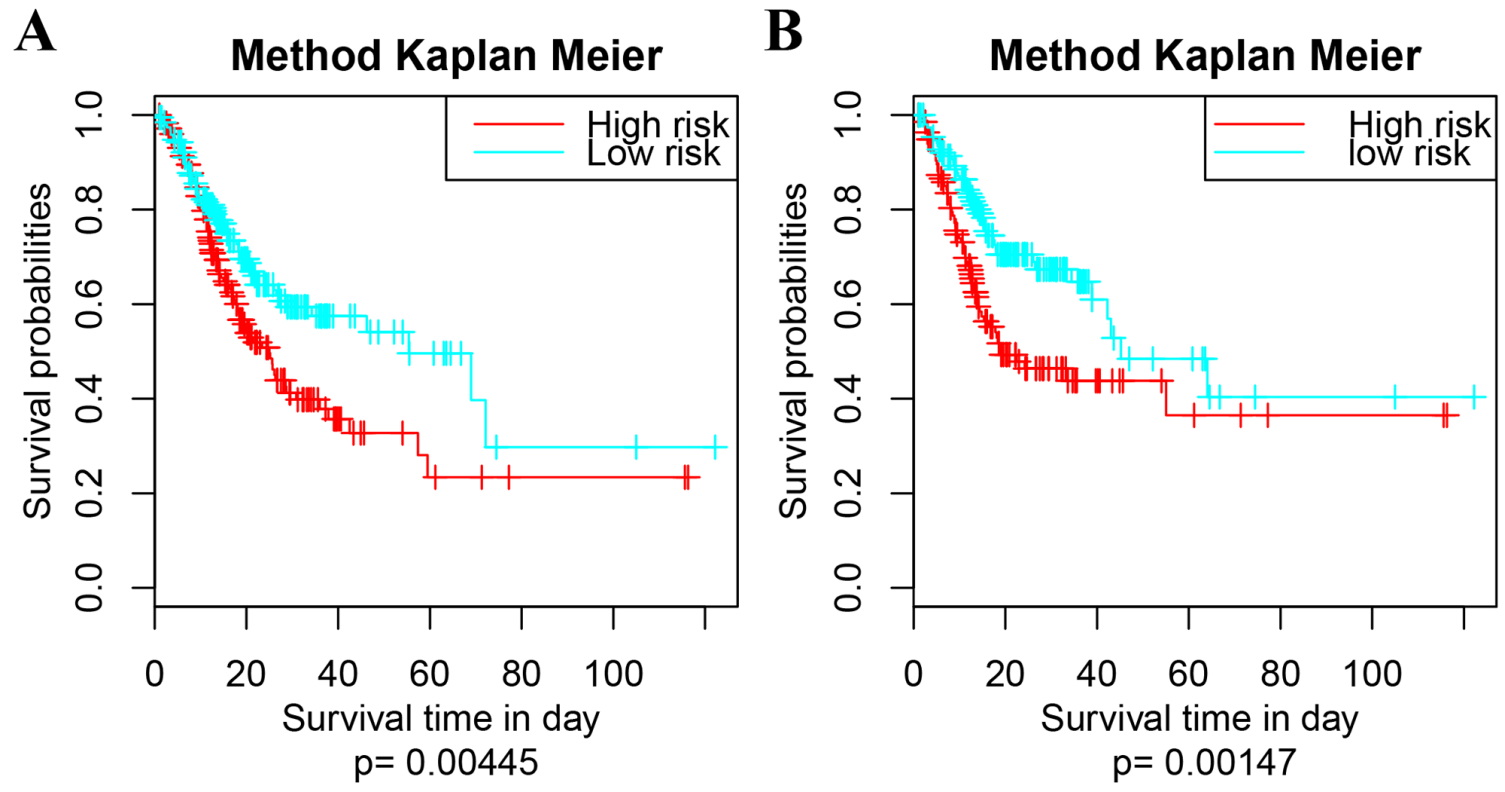
Survival analysis of the extra dataset following the 9-5Gene model **(A)** Kaplan Meier univariate survival analysis on survival time, p = 0.00445; **(B)** Kaplan Meier univariate survival analysis on re-occurrence risk, p = 0.00147. The survival time is calculated by day. The threshold is p value < 0.05.

The expressing profiles of these 9 feature genes were obtained from TCGA, basing on which 415 samples of GC patients were clustered into high- and low- risk group following the ≥5Genes mode. The Kaplan Meier univariate survival analysis of each cluster were shown in Figure 6. The difference in survival time of the GC patients between high- and low- risk group was significant (p=0.00445); and the difference in recurrence was as well significant (p=0.00147).

## DISCUSSION

Gastric cancer (GC) is a highly lethal malignancy with poor five-year survival rate comparing with any solid tumors, improving only modestly over the last 50 years, from 12 % to 22 % [13]. The remaining highly reoccurrence rate of GC patients leading to a worrying outcome. Accordingly, the evaluation of the prognosis of GC patients is essential. And identifying molecular biology of the individual tumor might be the key point to improve the prognosis of GC patients. It is true that much identification of various biomarkers has added to our basic knowledge of molecular and cellular mechanisms of GC tumorigenesis and progression [14]. Besides, basing on the expressing profiles of some genes influencing the prognosis of GC, the outcomes are able to be evaluated and predicted previously [15–18].

In many previous studies, gene signatures were ever identified when analyzing expressions in residual gastric cancer cells after neoadjuvant chemotherapy, composing of GSK3B, the β-catenin gene CTNNB1, NOTCH2 and many other genes [19, 20]. Besides, another type of prognostic signature, long non-coding RNA (lncRNAs) [21, 22] were as well identified to improve prognosis prediction of gastric cancer. Although these molecular characterization studies have attempted to identify the gene signature for prognosis in GC, signatures for the prognostic predicting system that is practically used in preclinical and clinical research are still inadequate.

In this study, the expressing profiles of GC were analyzed and the most significant feature genes were identified, with which a stable and effective 9-genes prognostic feature (NR1I2, LGALSL, C1ORF198, CST2, LAMP5, FOXS1, CES1P1, MMP7 and COL8A1) was constructed. To solve the over-fitting issues in this study, we go for simpler models over more complicated models using fewer parameters and remove one parameter without much increasing the (out-of-sample prediction) error.

To perform the validation process, we tried Cross-Validation, a standard way to find out-of-sample prediction error is to use 5-fold cross validation. In this case, we used 9 differentially expressed genes to build the model, and then evaluate the error on the 4 genes left out of the model. To repeat this 4 times, we used a different set of 5Gene each time. This is a better representative of the error. Combing with Kaplan Meier univariate and Robust likelihood-based survival analysis, as well as clustering analysis, the optimistic method to classify the GC patients into high- and risk- group was the no less than 5 genes model (≥5Genes).

Because the data in TCGA (The Cancer Genome Atlas) are generated by larger teams of research and more standardized than those in GEO, the predictive value basing on the TCGA cohort is less than that basing on the GEO cohort. To achieve an advanced molecular classifier and predictors, we are able the take good advantage of the large-scale profiling data in GC at TCGA. It was verified that the 9-5Gene clustering model can effectively classify patients from both the selected dataset and the extra one into high- and low- risk group with significant differences on survival time and reoccurrence risk.

In the previous studies, some of these 9 genes were identified differentially expressing in other cancer patients and that some were analyzed to explain the bio-functions. Hereinto, NR1I2 (nuclear receptor subfamily 1 group I member 2) was identified involved in the pharmacogenetics of irinotecan, which might help to predict the toxicity of low-dose irinotecan [23]. These Nuclear-Receptors represent candidates with potential oncogenic properties, including NR1I2, contributed to the development of therapeutic strategies for increasing their expression or activating them in tumor cells [24]. LGALSL has not been identified in any tumor till now. In this study, it is the first time to identify NR1I2 and LGALSL associated with gastric cancer as negative impact factors influencing the prognosis of this cancer.

In previous study, C1orf198 was identified as novel gene in colon, gastric and pancreatic cancer, however, the GO functions of this gene were unknown yet [25]. Gene CST2, belonging to cystatin gene (CST) family, was reported as a unique allele showing that amino acid substitution in one of the most conserved regions was responsible for cysteine proteinase inhibitory activity [26]. As well, studies indicated that in vivo bone metastasis was promoted by high expression of the salivary cystatins CST1, CST2, and CST4 [27]. LAMP5 (lysosome-associated membrane protein (LAMP)) has ever been identified among the 8 genes as a prognostic algorithm for gastric cancer (GCPS) that can robustly identify high-risk group for recurrence among stage II patients [28]. Similarly, MMP7 was ever screened out among novel plasma tumor markers, consisting of 11 genes [25]. It is also well-studied that the COL8A1 (collagen type VIII, alpha-1) gene, which encodes the alpha 1 chain of collagen, may modulate migration, proliferation and adherence of various cells. But a few studies also suggested that COL8A1 might represent a new potential target for gene therapy in hepatocarcinoma [29]. Taken together, this gene deserved more studies on its functional mechanism. Although the function and role of some genes in the 9-genes selected have not been reported for their association with GC, their importance as essential parts of the 9-gene signature could not be overlooked as well.

Traditionally, tumor node metastasis (TNM) staging is the common classification system. Besides, another two well-recognized classification systems: the Lauren classification, subdividing GC into intestine and diffuse types; and the alternative World Health Organization (WHO) system that divides gastric cancer into papillary, tubular, mucinous (colloid), and poorly cohesive carcinomas. However, comparing with the novel microarray technologies for GC subtypes by gene expression profiling [30], the prediction accuracy of these three methods are lower than the gene-signatures. Our survival analysis of the outcomes and reoccurrence are basing on the disease symptoms and diagnoses. By using similar methods, a 92-gene signature developed by Ken et al achieved an accuracy of 92%. [31], a 10-gene signature that was used to predict early stage had an accuracy of 90% and a 9-gene signature to predict cancer in stage III + IV made an accuracy of 84% [32]. The AUC of the 9-five gene signature in the present study is 0.741, which is regarded as high prediction accuracy. Besides, in term of the new biomarker (NR1I2) which might help to predict the toxicity of low-dose irinotecan, this classifier might achieve a higher accuracy in predicting the outcome of GC. Accordingly, the precise accuracy ratio can be calculate and compared with other classifiers in the further researches. In addition, gastric cancer was classified into four major genomic subtypes: EBV-infected tumors, MSI tumors, gnomically stable tumors, and chromosomally unstable tumors [33]. This classification is a valuable adjunct to histopathology and their genomic features are useful in clinical trials for definite GC patients. The 9-gene feature basing on the outcomes of patients reflects an overall prognosis of patients and can be used to predict the risks of un-distinct GC patients as well. Thus, 4 molecular subtypes could be a kind of complementary reference for the identified 9-gene feature is not related to histopathology directly.

In conclusion, the constructed 9-genes feature was effective and stable in classifying the samples from GC patients. And the ≥5Genes cluster model worked well in clustering patients into high- and low-risk groups with significant differences on both survival time and reoccurrence. Besides, most of the 9 genes were the first time finding associated with GC and can influencing the prognosis of patients. This feature gene model might contribute a lot to the development to predict the GC patients and help improve the outcomes by offering effective prognostic evaluation.

## MATERIALS AND METHODS

### Data source and processing

Microarray gene expression profiles of gastric cancer, GSE26253 [34], were downloaded from Gene Expression Omnibus database (GEO http://www.ncbi.nlm.nih.gov/geo/) for prognostic modeling on platform Illumina HumanRef-8 WG-DASL v3.0. Dataset in the Cancer Genome Atlas (TCGA) [35] were used to validate the prognostic model.

Standardized data of GSE26253 were downloaded and the probe-level data were converted into the corresponding genetic symbols to remove the non-matched probes. The corresponding RNA-sequencing data were obtained from cBioPortal in Cancer Genomics (http://www.cbioportal.org/), as well as the clinical follow-ups for validation of the constructed prognostic model.

### Differential analysis for prognostic genes

First of all, genes with significantly differential expressions among patients were screened as the follows: 1) the median expression level of this gene in every sample was more than 20% of the total median expressions of all genes in every sample; 2) the variance of expression level of one gene in every sample was higher than 20% of the total expression variances of all genes in every sample.

Then single-factor survival analysis of the differentially expressed genes was performed by using Survival in R Language [36]. Genes with significant p value < 0.05 were selected as seed genes.

### Robust likelihood-based survival modeling

For the utility of the seed genes in clinical diagnosis, Robust likelihood-based survival models [37, 38] were constructed to select feature genes by using rbsurv in R Language [38–40] as the followings:

1) The samples were randomly divided into the training set with N*(1 − p) samples and the validation set with N*p samples, with p = 1/3. In subsequence, a gene was fitted to the training set of samples, obtaining the parameter estimate for this gene. Then we evaluated log likelihood with the parameter estimate and the validation set of samples. This evaluation was repeated for each gene.

2) The above procedure was repeated for 10 times, thus we obtained 10 log-likelihood of each gene. The best gene, g (1), with the largest mean log likelihood was selected.

We searched the next best gene by evaluating every two-gene model and selected an ideal one with the largest mean log likelihood.

3) This forward gene selection procedure was continued resulting in a series of models. Akaike information criterions (AICs) for all the candidate models were computed and an optimal model with the smallest AIC was selected finally.

Hereinto, 300 samples were repeated in procedure of Robust likelihood-based survival model for 1000 times. The most frequent gene combinations were finally selected as final prognostic feature genes.

### Multivariate survival analysis of prognostic genes

Multivariate survival analysis of feature genes was performed to check the effect of the overall genes on the prognosis of gastric cancer. Estimated relative risks of poor prognosis were expressed as adjusted p value and corresponding 95 % confidence intervals (95 % CI). Multivariate survival analysis was used to evaluate the effect of each specific parameter. For multivariate analyses, Cox proportional hazards regression was applied. Then survival ROC [41] in R Language was used to finish the area under the curve (AUC) values for seed genes.

### Clustering analysis of prognostic genes

According to the expression profiles of the feature genes, each sample was classified by the unsupervised hierarchical clustering, then Kaplan Meier Survival analysis (http://kmplot. com/analysis/index.php?p=service&cancer=gastric) was performed to identify the prognostic differences between samples after classification. Meanwhile, the expressing differences between the profiles of feature genes and that of the normal tissues as well.

### Construction of prognostic model

Basing on the expressing patterns in the clustering analysis, genes were defined as positive-impacting factors (genes with positive in Figure 1) and negative-impacting factors (genes with negative in Figure 1), with which samples were classified again as follows:

1) The positive-impacting factors will be marked as active ones when their expressions were higher than the median.

2) The negative-impacting factors will be marked as active ones when their expressions were lower than the median.

3) Calculating the numbers of the active positive- or negative- impacting genes of every patient, patients were then classified according to the active numbers (≥1, ≥2, ≥3…).

Diversiform clustering algorithms were obtained, and then Kaplan Meier univariate survival analysis of these different algorithms was performed to calculate their influences on the prognosis of GC. Repetitive training of random samples in each model (samples contain at least a death event) to verify the model's stability. Finally, the optimistic clustering model was selected.

### Validation of the prognostic model

Selecting the ideal classification model, level 3 released gene level expression data of GC were downloaded from TCGA. The data processing and quality control were done by Broad Institute's TCGA workgroup. These patient samples of GC patients downloaded from another dataset were clustered into high- and low- risk group following the ≥5Genes mode. The Kaplan Meier univariate survival analysis was applied as well to compare the differences of the GC patients between high- and low- risk group in survival time and recurrence.
